# Significant role of PB1 and UBA domains in multimerization of Joka2, a selective autophagy cargo receptor from tobacco

**DOI:** 10.3389/fpls.2014.00013

**Published:** 2014-01-31

**Authors:** Katarzyna Zientara-Rytter, Agnieszka Sirko

**Affiliations:** Department of Plant Biochemistry, Institute of Biochemistry and Biophysics, Polish Academy of SciencesWarsaw, Poland

**Keywords:** Joka2, PB1, UBA, autophagy, proteasome, ubiquitin, selective autophagy cargo receptor, NBR1

## Abstract

Tobacco Joka2 protein is a hybrid homolog of two mammalian selective autophagy cargo receptors, p62 and NBR1. These proteins can directly interact with the members of ATG8 family and the polyubiquitinated cargoes designed for degradation. Function of the selective autophagy cargo receptors relies on their ability to form protein aggregates. It has been shown that the N-terminal PB1 domain of p62 is involved in formation of aggregates, while the UBA domains of p62 and NBR1 have been associated mainly with cargo binding. Here we focus on roles of PB1 and UBA domains in localization and aggregation of Joka2 in plant cells. We show that Joka2 can homodimerize not only through its N-terminal PB1-PB1 interactions but also via interaction between N-terminal PB1 and C-terminal UBA domains. We also demonstrate that Joka2 co-localizes with recombinant ubiquitin and sequestrates it into aggregates and that C-terminal part (containing UBA domains) is sufficient for this effect. Our results indicate that Joka2 accumulates in cytoplasmic aggregates and suggest that in addition to these multimeric forms it also exists in the nucleus and cytoplasm in a monomeric form.

## Introduction

Autophagy is a highly evolutionary conserved process among all eukaryotic organisms. It is responsible for degradation of cellular components in ubiquitin-proteasome system (UPS) independent manner (Yoshimori, 2004). The cellular components could be degraded by autophagy in unselective or selective manner. In the latter case the specific proteins, so called selective autophagy receptors, capable of the selective recognition of the cargos are needed (Weidberg et al., 2011). Soluble proteins, protein aggregates, or other cellular components assigned for degradation in the selective manner are usually marked by a polyubiquitin tail (Hershko and Ciechanover, 1998) which is recognized by the selective autophagy cargo receptors as a signal for degradation (Wilkinson et al., 2001). The selective autophagy cargo receptors control selectivity of autophagy flux. Similarly to other proteins involved in signaling and regulatory pathways they have modular domains responsible for specific interactions with variety of proteins (Pawson and Nash, 2003). Such form of regulation guarantees interconnections with the wide range of pathways and provides exact control of the appropriate process.

Both the N-terminal PB1 (Phox and Bem1) domains and the C-terminal UBA (ubiquitin associated) domains of p62 and NBR1 as well as of their homologs from animals, fungi, and plants are recognized as modules mediating protein-protein interaction (Geetha and Wooten, 2002; Kirkin et al., 2009a,b). Interestingly, p62 contains only one UBA domain, while NBR1 and plant selective autophagy cargo receptors, such as tobacco Joka2 and Arabidopsis AtNBR1 have two non-identical UBA domains. The animal proteins contain JUBA and UBA, while the plant proteins contain UBA1 and UBA2 domains. It has been shown that only UBA2 of AtNBR1 (NBR1 from Arabidopsis) can bind ubiquitin *in vitro* (Svenning et al., 2011). Both PB1 and UBA domains of p62 appeared absolutely crucial for its ability to form characteristic cytoplasmic bodies and for its function as a factor driving polyubiquitinated cargos to the autophagic degradation machinery. Therefore, specific degradation of polyubiquitinated cargos is highly dependent on two features of p62, its polymerization via the N-terminal PB1 domain and its ability to bind polyubiquitin via the C-terminal UBA domain (Bjorkoy et al., 2005).

PB1 domain is a protein interaction module conserved in animals, fungi, amoebas, and plants (Sumimoto et al., 2007). It was first found in phagocyte oxidase activator p67^phox^ and the yeast polarity protein Bem1p (Ito et al., 2001). According to the recent data, in all eukaryotes there are nearly 200 proteins containing the PB1 domain (Letunic et al., 2002). It is about 80 amino acids long and possesses an ubiquitin-like β-grasp fold containing two alpha helices and mixed five-stranded β-sheets. Additionally, it can harbor an OPCA (OPR/PC/AID) motif composed of about 20-amino acid with highly conserved acidic and hydrophobic residues and/or lysine residue conserved on the first β-strand (Ponting, 1996; Nakamura et al., 1998; Moscat and Diaz-Meco, 2000; Terasawa et al., 2001; Ponting et al., 2002). The PB1 domain present in mammalian p62 possesses both, the acidic OPCA motif and the conserved lysine (a residue of basic charge). It enables specific PB1-PB1 dimerization due to salt bridges formation between the OPCA from one PB1 and the lysine from the other PB1 (Gong et al., 1999; Sanz et al., 1999, 2000; Avila et al., 2002; Cariou et al., 2002; Lamark et al., 2003). The PB1 domain of p62 is responsible not only for homo-dimerization but also for interaction with other proteins. Conversely, the PB1 domain of mammalian NBR1 harbors only the OPCA motif and lacks the lysine residue what enables hetero-dimerization but is not sufficient for NBR1-NBR1 homo-dimers formation via PB1. Thus, additional CC motifs are involved in homo-dimerization of NBR1 proteins (Lamark et al., 2003). Interestingly, an ubiquitin fold of the PB1 domain is structurally similar to the ubiquitin and to the UbL (ubiquitin-like) domain and (Hirano et al., 2004). Although much weaker than the conventional ubiquitin-UBA binding, an apparent interaction between UbL and UBA domains of Dsk2 protein was indicated (Lowe et al., 2006). For those reasons it was postulated that the PB1 domain of p62 could be recognized by its UBA domain.

The UBA domain was initially identified by bioinformatic analysis (Hofmann and Bucher, 1996). It is about 45 residues long domain formed by three alpha helices and a hydrophobic patch mediating protein–protein interaction (Dieckmann et al., 1998). The UBA domain is found in many proteins involved in the degradation pathways engaging ubiquitin-like proteins, for example in Dsk2 or Rad23 involved in UPS or in p62 and NBR1 involved in autophagy-lysosomal machinery. Most UBA domains, but not all of them (Davies et al., 2004), are able to bind various ubiquitin forms, such as monoubiquitin or the K48- or K63-chains of polyubiquitin (Vadlamudi et al., 1996; Bertolaet et al., 2001a,b; Wilkinson et al., 2001; Funakoshi et al., 2002; Rao and Sastry, 2002). For instance, the UBA domain of p62 shows a preference for K63-polyubiquitinated substrates (Seibenhener et al., 2004; Long et al., 2008).

Although the mammalian p62 and NBR1 proteins were extensively studied, their plant homologs are far less characterized. Previously, it has been shown by us that Joka2, a selective autophagy cargo receptor from tobacco, is a functional and structural hybrid of mammalian selective autophagy cargo receptors by sharing some features of p62 and some of NBR1 (Zientara-Rytter et al., 2011). In this study we focused on two regions of Joka2, the N-terminal PB1 domain and the C-terminal region containing UBA domains. Our results pointed out their significant role in oligomerization and aggregation of Joka2 in plant cells.

## Materials and methods

### DNA cloning and plasmid construction

Plasmids used in this study are listed in Table 1. Details of their construction are available upon request. Sequences encoding recombinant unstable ubiquitin (Ub^G76V^) linked to YFP were designed based on previous results (Heessen et al., 2003). Gateway entry vectors were created by cDNA cloning into pENTR™/D-TOPO vector. Gateway LR recombination reactions were done as described in the Gateway® Technology—manual (Invitrogen, 12535-019 and 12535-027, respectively). Oligonucleotides for PCR and DNA sequencing are listed in Table 2. All plasmids were checked by DNA sequencing and/or by digestion by restriction enzymes. Conventional techniques were used for *Escherichia coli* or *Agrobacterium tumefaciens* transformation.

**Table 1:** **Plasmids used in this study**.

**Plasmid**	**Description**	**References**
**GATEWAY ENTRY VECTOR**		
pENTR-D TOPO	Entry vector for subcloning in gateway technology	Invitrogen
**GATEWAY DESTINATION VECTORS**		
pSITE-nEYFP-C1	Binary vector for BiFC	Chakrabarty et al., 2007; Martin et al., 2009
pSITE-cEYFP-C1	Binary vector for BiFC	Chakrabarty et al., 2007; Martin et al., 2009
pSITE-nEYFP-N1	Binary vector for BiFC	Chakrabarty et al., 2007; Martin et al., 2009
pSITE-cEYFP-N1	Binary vector for BiFC	Chakrabarty et al., 2007; Martin et al., 2009
pSITE-2CA	Binary vector for *gfp* fusion at N-terminus of cDNA	Chakrabarty et al., 2007
pSITE-4CA	Binary vector for r*fp* fusion at N-terminus of cDNA	Chakrabarty et al., 2007
pSITE-4NB	Binary vector for r*fp* fusion at C-terminus of cDNA	Chakrabarty et al., 2007
pH7CWG2	Binary vector for c*fp* fusion at C-terminus of cDNA	Karimi et al., 2005
pK7WGY2	Binary vector for y*fp* fusion at N-terminus of cDNA	Karimi et al., 2005
pH7YWG2	Binary vector for y*fp* fusion at C-terminus of cDNA	Karimi et al., 2005
pK7CWG2	Binary vector for c*fp* fusion at C-terminus of cDNA	Karimi et al., 2005
pH7WGC2	Binary vector for c*fp* fusion at N-terminus of cDNA	Karimi et al., 2005
pDEST22	“Prey” vector for Y2H with AD domain of GAL4 protein fused to cDNA N-terminus	Invitrogen
pDEST32	“Bait” vector for Y2H with BD domain of GAL4 protein fused to cDNA N-terminus	Invitrogen
**CONSTRUCTED GATEWAY ENTRY VECTORS FOR SUBCLONING**		
pEntrUBA	UBA domains (1444–2526 bp/482–842 aa) from NtJoka2 in pENTR-D TOPO	This study
pEntrUb-VV	NtUb^G76V^ in pENTR-D TOPO	This study
pEntrPB1	PB1 domain (1–1266 bp/1–422 aa) from NtJoka2 in pENTR-D TOPO	Zientara-Rytter et al., 2011
pEntrPB1ZZ	PB1ZZ domain (1–2253 bp/1–751 aa) from NtJoka2 in pENTR-D TOPO	Zientara-Rytter et al., 2011
pEntrZZ	ZZ domain (316–2253 bp/106–751 aa) from NtJoka2 in pENTR-D TOPO	Zientara-Rytter et al., 2011
pEntrZZUBA	ZZUBA domain (316–2526 bp/106–842 aa) from NtJoka2 in pENTR-D TOPO	Zientara-Rytter et al., 2011
pEntrATG8f	NtATG8f cDNA in pENTR-D TOPO	Zientara-Rytter et al., 2011
pEntrJ	Full-length NtJoka2 in pDONR221	Zientara-Rytter et al., 2011
U17036	ORF cDNA of AtNBR1 in vector pENTR/SD-TOPO for subcloning and direct expression	www.arabidopsis.org
**CONSTRUCTED PLANT EXPRESSION VECTORS**		
PB1-YFP	PB1 domain (1–1266 bp/1–422 aa) of NtJoka2 from pEntrPB1 in pH7YWG2	This study
PB1-CFP	PB1 domain (1–1266 bp/1–422 aa) of NtJoka2 from pEntrPB1 in pH7CWG2	This study
PB1ZZ-YFP	PB1-ZZ domains (1–2253 bp/1–751 aa) of NtJoka2 from pEntrPB1ZZ in pH7YWG2	This study
PB1ZZ-CFP	PB1-ZZ domains (1–2253 bp/1–751 aa) of NtJoka2 from pEntrPB1ZZ in pK7CWG2	This study
INT1-YFP	First interdomain region (316–1266 bp/106–422 aa) of NtJoka2 from pEntrINT1 in pH7YWG2	This study
INT2-YFP	Second interdomain region (−2253 bp/–751 aa) of NtJoka2 from pEntrINT2 in pH7YWG2	This study
ZZ-YFP	ZZ domain (316–2253 bp/106–751 aa) of NtJoka2 from pEntrZZ in pH7YWG2	This study
ZZUBA-YFP	ZZ-UBA domains (316–2526 bp/106–842 aa) of NtJoka2 from pEntrZZUBA in pH7YWG2	This study
ZZUBA-CFP	ZZ-UBA domains (316–2526 bp/106–842 aa) of NtJoka2 from pEntrZZUBA in pH7CWG2	This study
CFP-ZZUBA	ZZ-UBA domains (316–2526 bp/106–842 aa) of NtJoka2 from pEntrZZUBA in pH7WGC2	This study
UBA-YFP	UBA domains (1444–2526 bp/482–842 aa) of NtJoka2 from pEntrUBA in pH7YWG2	This study
UBA-CFP	UBA domains (1444–2526 bp/482–842 aa) of NtJoka2 from pEntrUBA in pH7CWG2	This study
Joka2-YFP	Full-length NtJoka2 from pEntrJ in pH7YWG2	This study
YN-ATG8f	Full-length NtATG8f from pEntrATG8f in pSITE-nEYFP-C1	This study
Joka2-YC	Full-length NtJoka2 from pEntrJ in pSITE-cEYFP-N1	This study
Joka2-YN	Full-length NtJoka2 from pEntrJ in pSITE-nEYFP-N1	This study
YC-Joka2	Full-length NtJoka2 from pEntrJ in pSITE-cEYFP-C1	This study
YN-Joka2	Full-length NtJoka2 from pEntrJ in pSITE-nEYFP-C1	This study
PB1ZZ-YN	PB1-ZZ domains (1–2253 bp/1–751 aa) of NtJoka2 from pEntrPB1ZZ in pSITE-nEYFP-N1	This study
PB1ZZ-YC	PB1-ZZ domains (1–2253 bp/1–751 aa) of NtJoka2 from pEntrPB1ZZ in pSITE-cEYFP-N1	This study
PB1-YC	PB1 domain (1–1266 bp/1–422 aa) of NtJoka2 from pEntrPB1 in pSITE-cEYFP-N1	This study
PB1-YN	PB1 domain (1–1266 bp/1–422 aa) of NtJoka2 from pEntrPB1 in pSITE-nEYFP-N1	This study
YC-PB1	PB1 domain (1–1266 bp/1–422 aa) of NtJoka2 from pEntrPB1 in pSITE-cEYFP-C1	This study
YN-PB1	PB1 domain (1–1266 bp/1–422 aa) of NtJoka2 from pEntrPB1 in pSITE-nEYFP-C1	This study
UBA-YC	UBA domains (–2526 bp/–842 aa) of NtJoka2 from pEntrUBA in pSITE-cEYFP-N1	This study
UBA-YN	UBA domains (–2526 bp/–842 aa) of NtJoka2 from pEntrUBA in pSITE-nEYFP-N1	This study
YC-UBA	UBA domains (–2526 bp/–842 aa) of NtJoka2 from pEntrUBA in pSITE-cEYFP-C1	This study
YN-UBA	UBA domains (–2526 bp/–842 aa) of NtJoka2 from pEntrUBA in pSITE-nEYFP-C1	This study
YC-NBR1	Full-length NtJoka2 from pEntrJ in pSITE-cEYFP-C1	This study
YN-NBR1	Full-length NtJoka2 from pEntrJ in pSITE-nEYFP-C1	This study
Joka2-RFP	Full-length NtJoka2 from pEntrJ in pSITE-4NB	This study
GFP-NBR1	Full-length NtJoka2 from pEntrJ in pSITE-2CA	This study
RFP-NBR1	Full-length NtJoka2 from pEntrJ in pSITE-4CA	This study
Ub-VV-YFP	NtUb from pEntrUb-VV in pH7YWG2	This study
**CONSTRUCTED YEAST EXPRESSION VECTORS**		
AD-UBA	UBA domains (1444–2526 bp/482–842 aa) of NtJoka2 from pEntrUBA in pDEST22	This study
BD-UBA	UBA domains (1444–2526 bp/482–842 aa) of NtJoka2 from pEntrUBA in pDEST32	This study
AD-PB1	PB1 domain (1–1266 bp/1–422 aa) of NtJoka2 from pEntrPB1 in pDEST22	Zientara-Rytter et al., 2011
BD-PB1	PB1 domain (1–1266 bp/1–422 aa) of NtJoka2 from pEntrPB1 in pDEST32	Zientara-Rytter et al., 2011
AD-PB1ZZ	PB1-ZZ domains (1–2253 bp/1–751 aa) of NtJoka2 from pEntrPB1ZZ in pDEST22	Zientara-Rytter et al., 2011
BD-PB1ZZ	PB1-ZZ domains (1–2253 bp/1–751 aa) of NtJoka2 from pEntrPB1ZZ in pDEST32	Zientara-Rytter et al., 2011
AD-ZZUBA	ZZ-UBA domains (316–2526 bp/106–842 aa) of NtJoka2 from pEntrZZUBA in pDEST22	Zientara-Rytter et al., 2011
BD-ZZUBA	ZZ-UBA domains (316–2526 bp/106–842 aa) of NtJoka2 from pEntrZZUBA in pDEST32	Zientara-Rytter et al., 2011
**YEAST EXPRESSION VECTORS USED AS A CONTROLS**		
pEXP32/Krev1	Yeast expression vector to use as a “bait” for interaction strength controls	Invitrogen
pEXP22/RalGDS-wt	Yeast expression “prey” vector for strong interaction control	Invitrogen
pEXP22/RalGDS-m1	Yeast expression “prey” vector for weak interaction control	Invitrogen
pEXP22/RalGDS-m2	Yeast expression “prey” vector for negative interaction control	Invitrogen
**PLANT EXPRESSION VECTORS USED AS A LOCALIZATION CONTROLS**		
vac-ck CD3-969	Tonoplast marker—binary plasmid with a CFP fuses to the C-terminus of γ-TIP, an aquaporin of the vacuolar membrane	Nelson et al., 2007

**Table 2:** **Oligonucleotides used for PCR and DNA sequencing**.

**Name**	**Sequence**	**Description**
Joka2-F3	caccatgaagggtttacatgatct	For cloning UBA domains with second interdomain region of Joka2
Joka2-R3	ctctccagcaataagatccatg	
Joka2-F2	caccatgtctactcccttacgatc	For cloning first interdomain region of Joka2
Joka2-R1	aatagtcccagtcccatcactg	
Joka2-F3	caccatgaagggtttacatgatct	For cloning second interdomain region of Joka2
Joka2-R2	ctggggtggtgcctgcg	
ubq-F	caccatgcagatcttcgtgaa	For cloning tobacco ubiquitin cDNA
ubq-R-VV	cttaccaacaacaccacggagacggaggac	
att-L2	gtacaagaaagctgggtcg	For sequencing destination vectors from 3′ end
CaM35S-F	gatatctccactgacgtaagggatg	For sequencing binary vectors from 5′ end

### Yeast two hybrid assay

Yeast cells transformation was performed by the LiAc/ss carrier DNA/PEG method (Gietz and Woods, 2002) following the “Quick and Easy TRAFO Protocol.” After the transformation cells were placed on the appropriate synthetic dropout (SD) medium, prepared according to Invitrogen Handbook (PT3024-1), for transformants selection and, later, for testing of the possible protein-protein interactions. Plates were incubated at 30°C for up to 7 days.

### Plant material and growth conditions

*Nicotiana benthamiana* plants were grown in soil in growth chamber under the conditions of 60% relative humidity, with a day/night regime of 16 h light 300 μmol photons m^2−1^ s^−1^ at 23°C and 8 h dark at 19°C.

### Transient protein expression

For transient co-expression of proteins in *N. benthamiana* leaves fresh overnight cultures of *A. tumefaciens* containing appropriate binary plasmids were spun down and washed twice. Next, cells were re-suspended in sterile water and brought to a final cell density 2 × 10^8^ cfu/ml (OD600 ~ 0.2). For bimolecular fluorescent complementation (BiFC) experiments the cell suspensions were adjusted to 4 × 10^8^ cfu/ml and mixed 1:1 before infiltration. Young *N. benthamiana* plants with fully expanded leaves of about 5 cm in diameter were infiltrated by bacterial suspension using a needless syringe. Leaves were harvested and analyzed under confocal microscope 3 days after agroinfiltration.

### Confocal microscope analysis

For staining of nuclei, prior the microscope analysis, agroinfiltrated leaves were incubated with a fluorescent dye DAPI (1 μg/ml) for 15 min in the darkness at room temperature. After the treatment, plant material was washed in water (3 times, 5 min each) and immediately observed in a confocal microscope. For LMB treatment plant material was incubated with leptomycin B (20 ng/ml) up to 24 h before observation. All images were obtained in the Laboratory of Confocal and Fluorescence Microscopy at IBB PAS using a Nicon confocal microscope, Eclipse TE2000-E and processed using EZ-C1 3.60 FreeViewer software. For GFP/YFP the 488-nm line from an Argon-Ion Laser (40 mW) was used for excitation, and a 500–530 nm band pass filter for detection of emission. For RFP the 543 nm line of a Green He-Ne Laser (1.0 mW) was used for excitation and the 565–640 nm filter was used for detection. The same 543 nm line of a Green He-Ne Laser (1.0 mW) but with a 650 nm long pass filter was used for chlorophyll emission and detection, respectively. The blue fluorescence of DAPI or CFP was imaged using 404 nm Violet-Diode Laser MOD (44.8 mW) for excitation and 430–465 nm or 435–485 nm bands pass filter for emission.

## Results

### Joka2 localization and interactions in plant cells

**Figure 1:**
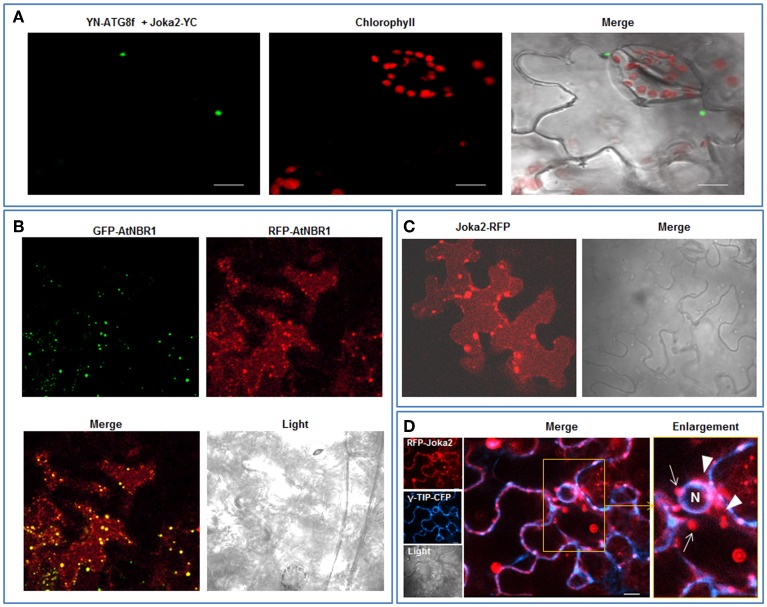
**Cytoplasmic and vacuolar localization of transiently expressed Joka2 and AtNBR1 in leaf epidermal cells of *N. benthamiana*. (A)** BiFC assay of interaction between Joka2 and NtATG8f using randomly chosen combination (YN-NtATG8f+Joka2-YC) of the vectors. **(B)** Co-localization of co-expressed GFP-AtNBR1 and RFP-AtNBR1 (AtNBR1 fused to two variants of fluorescent protein). **(C)** Localization of Joka2-RFP in the vacuole. **(D)** Subcellular localization of co-expressed RFP-Joka2 and γ-TIP-CFP—a tonoplast marker based on an aquaporin of the vacuolar membrane fused to CFP. The enlarged part of the picture visualizes tonoplast (the blue fluorescence signal) of the central vacuole which surrounds the nucleus (N) and red fluorescence of RFP-Joka2 fusion protein observed mainly inside the vacuole close to the tonoplast as a smear (arrowheads) or in spots (arrows). Scale bar, 10 μm.

**Figure 2:**
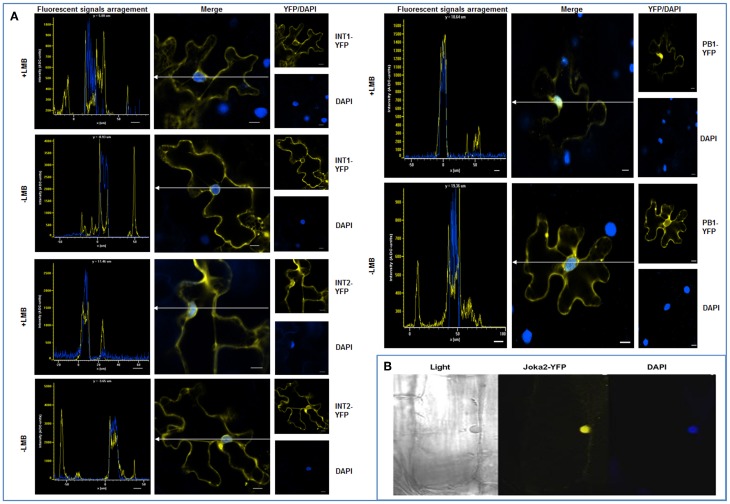
**Nuclear localization of Joka2. (A)** Subcellular localization of transiently expressed truncated Joka2 proteins (INT1-YFP, INT2-YFP, and PB1-YFP) in leaf epidermal cells of *N. benthamiana* treated (+LMB) and not treated (−LMB) with the inhibitor of nuclear export. The localization of INT1-YFP and the INT2-YFP was unaffected by the LMB treatment, while PB1-YFP remained in the nucleus only after treatment with LMB. White lines with arrows indicate the cross-section of the cells used in analysis shown to the left. The nuclei are stained blue with DAPI. **(B)** An rhizodermis cell of transgenic tobacco line J4-1 expressing Joka2-YFP (yellow) and DAPI staining (blue) indicating the nuclear localization of Joka2-YFP. Scale bar, 10 μm.

**Figure 3:**
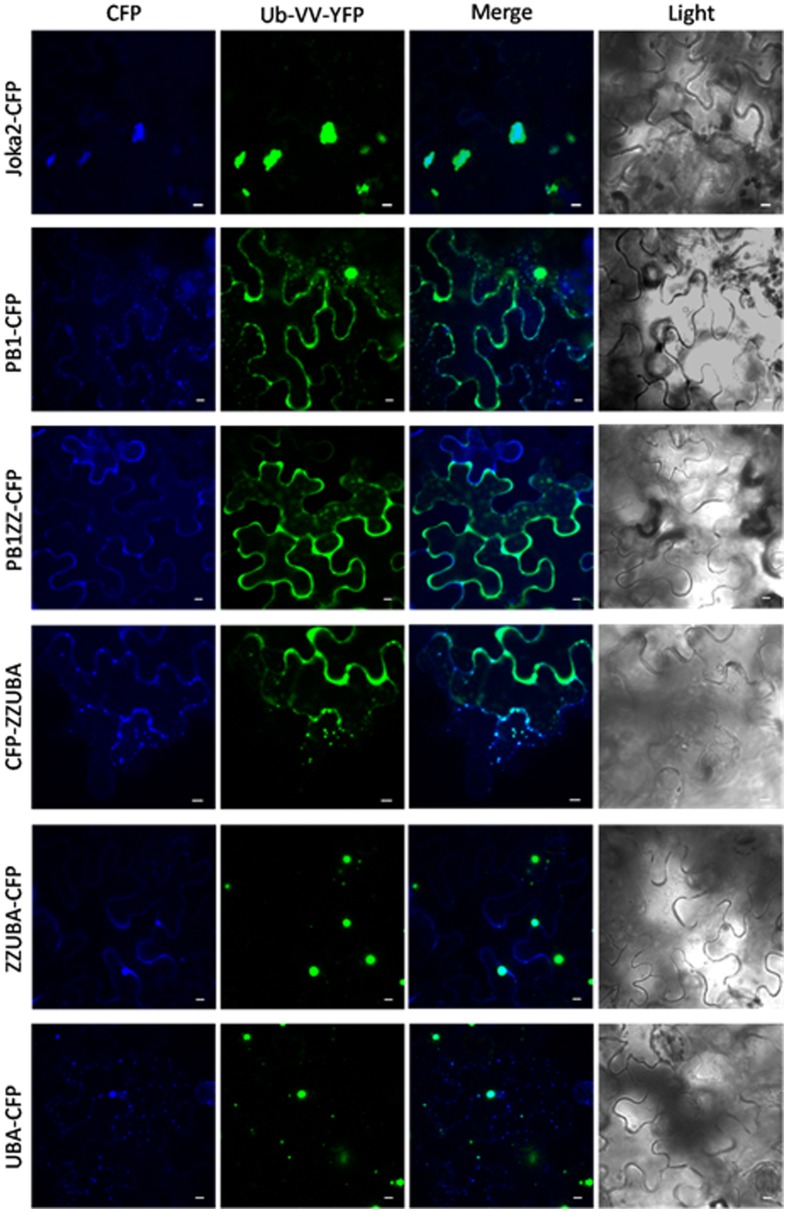
**Truncated Joka2 containing only UBA domains co-localizes with ubiquitin linked to YFP (Ub-VV-YFP)**. Truncated Joka2 proteins lacking PB1, PB1, and ZZ, ZZ, and UBA or UBA domains were transiently co-expressed in *N. benthamiana* leaves with unstable ubiquitin linked to YFP (Ub-VV-YFP). The overlapping fluorescent signals were observed only in the case of co-expression of Ub-VV-YFP with the following versions of the recombinant proteins: full-length Joka2-CFP, ZZUBA-CFP, CFP-ZZUBA, UBA-CFP. Scale bar, 10 μm.

Joka2 protein is a homolog of two human receptors of selective autophagy, p62 and NBR1. Similarly to these proteins, Joka2 not only forms small cytosolic, punctuated bodies which are imported to the central vacuole by autophagy machinery but also creates larger cytoplasmic aggregates. Also alike p62, Joka2 has been observed by us in a nucleus in stably transformed *Nicotiana tabacum* plants (Zientara-Rytter et al., 2011). To understand the phenomenon of this variable localization of Joka2 several plasmids encoding truncated variants of the protein were prepared (Figure S1). A series of co-localization experiments in leaves of *N. benthamiana* plants transiently transformed with the plasmids containing plant expression cassettes encoding various combinations of the fusion proteins were performed (Figures 1–3). Previously, localization of Joka2 in acidic speckles and co-localization of Joka2 with NtATG8f was established in our laboratory (Zientara-Rytter et al., 2011). The BiFC method, used in this study, confirmed not only the autophagosomal localization of Joka2 but also its direct *in vivo* interaction with NtATG8f (Figure 1A). Moreover, the vacuolar localization of Joka2 fused to RFP (Figures 1C,D) and the partial co-localization of the GFP-AtNBR1 and RFP-AtNBR1 proteins used as a localization control (Figure 1B) are in agreement with results reported previously for EGFP-mCherry-AtNBR1 suggesting that AtNBR1 is transported to the vacuole (Svenning et al., 2011). Additionally, the nuclear localization of Joka2 in stably transformed Joka2-YFP seedlings was confirmed by DAPI staining (Figure 2B) and the functionality of the nuclear export sequence (NES) located in the first interdomain region (INT1), between PB1 and ZZ domains was demonstrated (Figure 2A). The treatment with nuclear export inhibitor (LMB) enclosed the truncated PB1-YFP protein in the nucleus but did not change the cytoplasmic localization of the INT1 protein nor the localization of the INT2 protein, each fused to YFP (Figure 2A). The observed subcellular localization of INT1 and PB1 truncated proteins in the absence of LMB strongly suggests that the nuclear localization signal (NLS) is located in PB1 domain and not in INT1 region. The subcellular location of the truncated PB1-YFP protein is affected by LMB treatment similarly as the subcellular location of the full length Joka2 (Zientara-Rytter et al., 2011 and Figure S2), however LMB does not change intracellular distribution of the free green fluorescent protein (GFP) in stably transformed plants (Figure S3).

Finally, co-localization of Joka2-CFP with unstable recombinant ubiquitin linked to YFP (Ub-VV-YFP) indicated that Joka2 is present in ubiquitin-containing protein aggregates (Figure 3). It could be assumed that at least one of UBA domains of Joka2 is involved in recognition of ubiquitin-containing proteins and in sequestration of poly-ubiquitinated proteins into aggregates. To verify this assumption, the cassettes: PB1-CFP, PB1ZZ-CFP, ZZUBA-CFP, CFP-ZZUBA, UBA-CFP were transiently co-expressed in *N. benthamiana* leaves with the cassette for Ub-VV-YFP. This experiment showed that the C-terminal part of Joka2 possessing UBA domains was necessary for co-localization of Joka2 with Ub-VV-YFP. Moreover, the sequestration of Ub-VV-YFP into aggregates took place only in the presence of C-terminal UBA domains of Joka2, whereas N-terminal PB1 domain of Joka2 had no effect on its localization. Moreover, truncated Joka2 with ZZUBA or UBA domains were always co-localized with Ub-VV-YFP.

### Joka2 exists *in planta* in monomeric and in oligomeric forms

**Figure 4:**
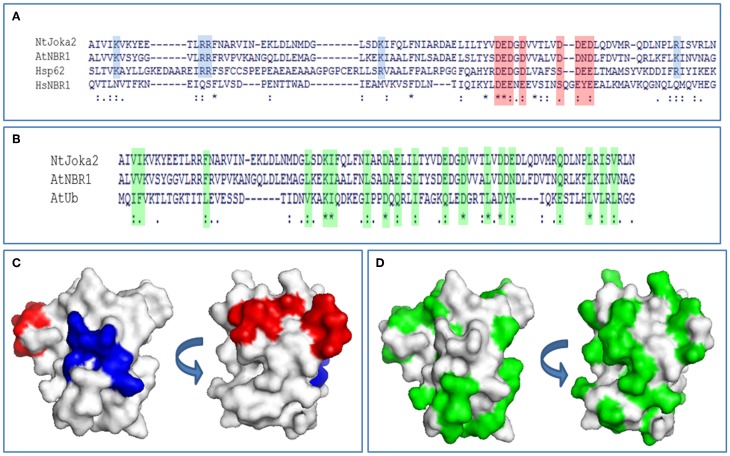
**PB1 sequence analysis. (A)** Alignment of PB1 domain sequences from tobacco Joka2, Arabidopsis NBR1, and *Homo sapiens* p62 and NBR1. Blue background color denotes basic residues and red background color denotes acidic residues from OPCA-motif important for PB1 domain interactions and self-interaction. **(B)** Alignment of PB1 domain sequences of Joka2, AtNBR1, and ubiquitin sequence from *Arabidopsis thaliana*. Green background color denotes similar residues between ubiquitin and PB1 domain of Joka2 and AtNBR1. Identical amino acids are indicated with asterisks and by dots are marked amino acids with high similarity. **(C)** PB1 domain from Joka2 modeled using Swissmodel (PBD: 2KKC). By blue color are marked basic residues and by red are colored acidic residues from OPCA-motif. Two surfaces are shown. **(D)** PB1 domain from Joka2 modeled using Swissmodel (PBD: 2KKC). Green color marks amino acids similar between ubiquitin and PB1 domain of Joka2. Two surfaces are shown.

**Figure 5:**
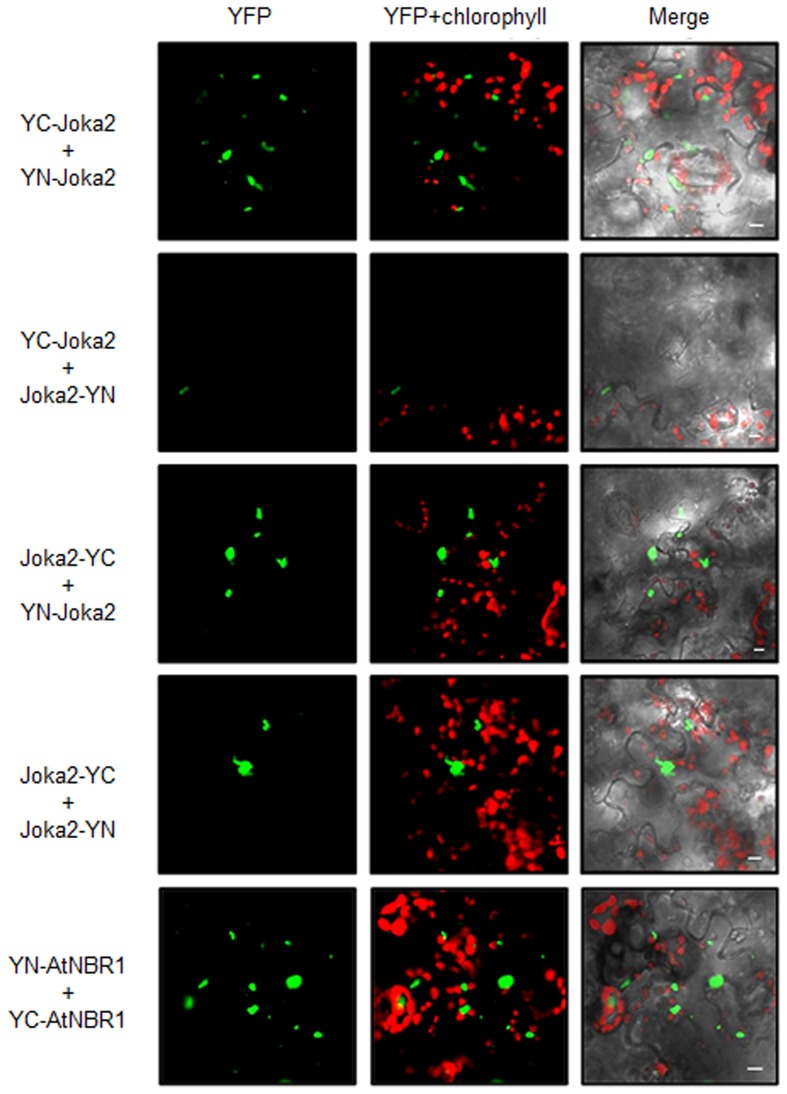
**BiFC assay of dimerization of transiently expressed Joka2 and AtNBR1 in leaf epidermal cells of *N. benthamiana***. *Four* combinations of BiFC plasmids were used for analysis of Joka2 dimerization (YC-Joka2+YN-Joka2, YC-Joka2+Joka2-YN, Joka2-YC+Joka2-YN, and Joka2-YC+YN-Joka2). Joka2-Joka2 interaction (green signal) was observed in all combinations. Dimerization of AtNBR1 was tested by BiFC method using only one randomly chosen plasmids combination (YC-AtNBR1+YN-AtNBR1). Scale bar, 10 μm. Negative controls are shown in Figure S5.

Due to a structural similarity between Phox/Bem1p (PB1) domain and ubiquitin-like (UbL) domain, selective autophagy cargo receptors (Joka2, p62, and NBR1) might be included into a family of ubiquitin receptor proteins containing both UbL and ubiquitin-associated (UBA) domains (Figures 4B,D). Moreover, p62 possess many properties that are similar to the UbL–UBA proteins, such as direct interaction with proteasome by PB1 domain (Babu et al., 2005; Geetha et al., 2008) and ability to deliver polyubiquitinated proteins to UPS (Seibenhener et al., 2004; Babu et al., 2005). It was shown by us previously in yeast two hybrid (Y2H) experiments that Joka2 can form homodimers (Zientara-Rytter et al., 2011). Here, Joka2 dimerization is confirmed *in planta* using the BiFC method in which fusion proteins linking Joka2 with either N- or C-terminal part of YFP (YN or YC, respectively; see Figure S4) were generated by transient co-expression in *N. benthamiana* leaves using all four possible combinations, namely YN-Joka2 with YC-Joka2, YN-Joka2 with Joka2-YC, Joka2-YN with Joka2-YC, and Joka2-YN with YC-Joka2 (Figure 5 and Figure S5). Additionally, self-interaction of AtNBR1, an Arabidopsis homolog of Joka2, was verified using one randomly selected combination, namely YN-AtNBR1 and YC-AtNBR1 (Figure 5). The BiFC assay confirmed that both cargo receptors, Joka2 and AtNBR1, were able to make multimeric forms *in planta*. Interestingly, for both proteins the fluorescence of the restored YFP was observed only in aggregates what suggests that Joka2 and AtNBR1 are present in oligomeric forms only in aggregasomes, while outside of aggregasomes, in the cytoplasm and the nucleus they rather exist in monomeric forms.

### PB1-PB1 interactions are sufficient for aggregates formation

It is obvious that aggregation of selective autophagy cargo receptors is possible due to their ability to polymerize. Molecular modeling of PB1 domain of Joka2 revealed that it has a basic/acidic surface structure, which is similar to PB1 domain of p62 which, in turn, has the ability to polymerize (Svenning et al., 2011). In the PB1 domain, both proteins harbor the N-terminal basic charge cluster and the C-terminal, acidic OPCA motif (Figures 4A,C).

**Figure 6:**
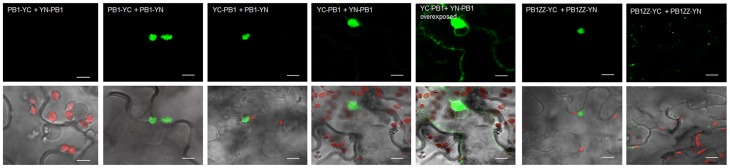
**BiFC assay of dimerization of PB1 *in planta***. The combinations of plasmids (PB1-YC+YN-PB1, PB1-YC+PB1-YN, YC-PB1+PB1-YN, YC-PB1+YN-PB1, and PB1ZZ-YC+PB1ZZ-YN) were used for BiFC analysis in leaf epidermal cells of *N. benthamiana*. The interaction (green signal) was mainly observed in cytosolic aggregates. For the combination of PB1-YC+YN-PB1 no fluorescence signal was observed in plant cells. For the combination of YC-PB1+YN-PB1 the weak fluorescence in cytoplasm was also present. Two independent representative pictures are shown for the combination of YC-PB1ZZ+YN-PB1ZZ. Scale bar, 10 μm.

Previously, it has been shown by us in Y2H experiments that the N-terminal PB1 domain of Joka2 is involved in dimers formation (Zientara-Rytter et al., 2011). We decided to confirm this result *in planta* by BiFC. The constructs encoding the PB1ZZ and PB1 fragments of Joka2 were used in this experiment. The fusion proteins were generated by linking PB1ZZ or PB1 with either N- or C-terminal parts of YFP and fluorescence of YFP was observed in several combinations of the fusions. For PB1ZZ only one combination was tested (PB1ZZ-YC+PB1ZZ-YN), while for PB1 all four combinations were used. The fluorescence was observed in all analyzed combinations except PB1-YC+YN-PB1 (Figure 6) and the negative controls (not shown). These results indicate that PB1 domain of Joka2 protein can form homo-dimers *in planta*.

### UBA domains are also involved in aggregasomes formation

**Figure 7:**
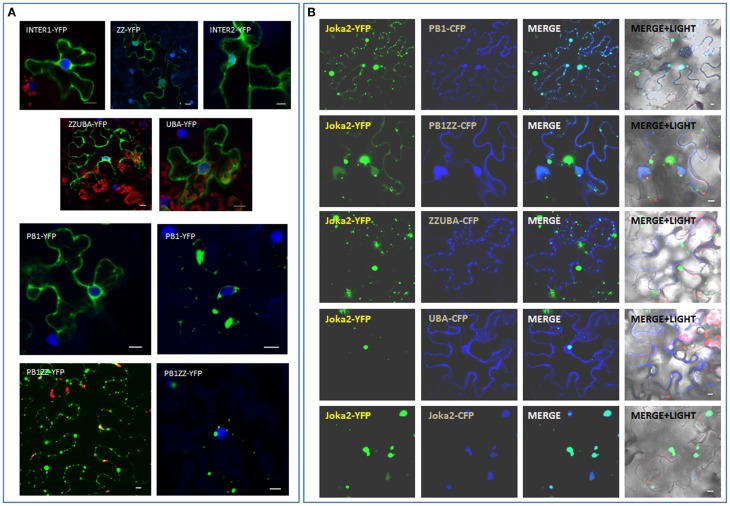
**Involvement of PB1 and UBA domains in formation of Joka2-Joka2 aggregates *in planta*. (A)** Localization of truncated forms of Joka2 in *N. benthamiana* epidermal cells. **(B)** Joka2 subcellular localization analysis after co-expression of Joka2-YFP with various truncated forms of Joka2 linked to CFP in *N. benthamiana* leaves. Scale bar, 10 μm.

During subsequent analysis, various Joka2 fragments linked to YFP or CFP were tested for their subcellular localization and ability to form cytoplasmic aggregates. Cassettes encoding the respective fusion proteins were transiently expressed in *N. benthamiana* leaves and the recombinant proteins were analyzed under confocal microscopy (Figure 7). Interestingly, somewhat diffused distribution was observed in each of the tested deletion constructs, namely PB1, PB1ZZ, INT1, ZZ, INT2, ZZUBA, UBA. Such distribution was similar to K11A/D60A mutant with disturbed acidic/basic surface described by the Johansen's group (Svenning et al., 2011). The truncated proteins containing PB1 domain (PB1, PB1ZZ) were able to create aggregates *in planta*, but this tendency was weaker (e.g., smaller and less aggregates and more apparent “diffused” distribution in the cytoplasm) than in the case of full Joka2 containing all three domains, PB1, ZZ, and double UBA (Figure 7A). In summary, this experiment indicated (i) necessity of PB1 domain for protein multimerization *in vivo* and (ii) contribution of UBA domains to the process of aggregates formation (or stabilization).

This problem was investigated further by transient co-production of the full length Joka2 (Joka2-YFP) with truncated versions (PB1 or PB1ZZ, ZZUBA, UBA) linked to CFP, what enabled monitoring of both types of the proteins in one cell (Figure 7B). Interestingly, full-length Joka2 co-expressed with some truncated forms (PB1 or PB1ZZ) was observed not only in aggregates but also a weak fluorescence was present in the nucleus and cytoplasm (Figure 7B). Such dual localization was not observed when Joka2 was co-produced with ZZUBA or UBA domains or with full-length Joka2 (Figure 7B). This result is in agreement with the results shown in Figures 3, 7A and indicates that UBA domains are also involved in cytoplasmic bodies formation. Moreover, this result strongly suggested a possibility of PB1-UBA interaction.

### PB1-UBA interactions in aggregasomes

**Figure 8:**
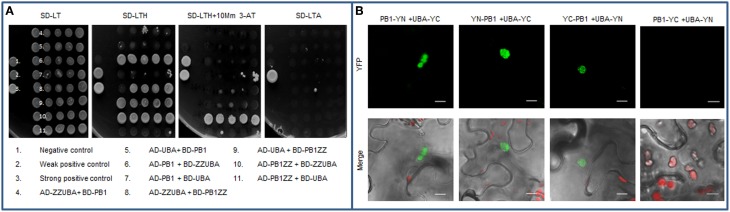
**Interaction between PB1 and UBA domains. (A)** Yeast two-hybrid (Y2H) analysis demonstrating weak interaction between PB1 domain and the fragment containing UBA domains of Joka2. Truncated proteins lacking either PB1 or UBA domains were fused to AD or BD domain of GAL4 protein and co-expressed in yeast cells (AH109 strain). Positive and negative controls for protein interaction analysis were provided by Invitrogen. **(B)** BiFC assay for PB1-UBA interaction *in planta*. Indicated combinations of plasmids (PB1-YN+UBA-YC, YN-PB1+UBA-YC, YC-PB1+UBA-YN, PB1-YC+UBA-YN) were used for analysis. Scale bar, 10 μm.

It is known that PB1 domains are also able to interact with other domains. For example, PB1 domain from p62 can directly interact with PB1 domain from NBR1 protein or with the Rpt1 subunit of 26S proteasome (Seibenhener et al., 2004; Babu et al., 2005; Geetha et al., 2008). The NMR studies of PB1 domain has shown that it creates an ubiquitin-like, β-grasp fold, similar to the well-characterized UbL domain (Hirano et al., 2004). Therefore, it was postulated that PB1 domain can also directly interacts with UBA (Su and Lau, 2009; Isogai et al., 2011). A similar interaction was reported for the UbL and UBA family of ubiquitin binding proteins involved in proteasomal degradation of ubiquitinated substrates, like Dsk2 protein (Lowe et al., 2006). Despite the fact that UBA domains were not crucial for multimerization of the selective autophagy cargo receptors, we decided to test if a direct interaction between PB1 and UBA domains from Joka2 is possible. The screening performed in Y2H system indicated that the interaction between these domains could take place *in vivo* (Figure 8A). The strongest interaction was observed when the truncated PB1ZZ protein was fused to AD domain of GAL4, while the truncated ZZUBA protein was fused to BD domain of GAL4. Nevertheless, it was still moderate interaction in comparison to the positive control and the other previously described by us interactions using Y2H system, namely PB1-PB1. Nevertheless, the PB1-UBA interaction was also confirmed by BiFC experiment *in planta*. Interestingly, the fluorescent signal from YFP (obtained as a consequence of a direct binding of UBA and PB1) was observed only in aggregates (Figure 8B) despite the fact that both fusion proteins share diffuse localization in *N. benthamiana* cells (see Figures 3, 7B).

## Discussion

The main focus of this study was on characterization of the role of PB1 and UBA domains in multimerization and aggregation of Joka2 in plant cells. The results are shown in a form of a model summarizing and explaining detected interactions (Figure 8). It has been shown by us that Joka2 has multiple cellular localizations. We have observed Joka2 in autophagosomes, where its interaction with ATG8 proteins is possible; in vacuole where it is presumably degraded; in cytosolic aggregates (aggregasomes) and in the nucleus. The nuclear location was especially apparent after treatment with LMB, an inhibitor of nuclear export. We were able to localize the functional NES (nuclear export sequence), however, localization of NLS (nuclear localization sequence) was not yet done. Several positions might be considered since several NLS consensuses were detected within Joka2. Nonetheless, the results shown in Figure 2A suggest that NLS must be present in the INT2 region. The function of Joka2 in nucleus is still unknown. It is possible that Joka2, similarly to ATG8 proteins, accumulates in the nucleus to prevent activation of autophagy by the excess of Joka2 in the cytoplasm or, since UPS is the main degradation pathway in the nucleus, Joka2 could be involved in shuttling of the protein cargos to the nuclear proteasomes as it is postulated for p62 (Pankiv et al., 2010). Such role of Joka2 is plausible due to the strong structure similarity of Phox/Bem1p (PB1) domain to the ubiquitin-like (UbL) domain directly interacting with proteasome compounds (Hirano et al., 2004).

Joka2 is a strongly aggregating protein. PB1 domain of Joka2 has similar basic/acidic surface to PB1 of p62 protein (Svenning et al., 2011; Zientara-Rytter et al., 2011). It is known that the N-terminal basic charge cluster is able to bind non-covalently to the C-terminal acidic OPCA motif. Our results indicate that PB1 domain is sufficient for Joka2 oligomerization *in planta* and that the C-terminal region containing UBA1 and UBA2 domains additionally promotes Joka2 aggregation. Moreover, the aggregates formed by the truncated proteins lacking the fragment with UBA domains are mostly not co-localized with ubiquitin aggregates (Figure 3), what is in agreement with previously proved involvement of UBA domains in recognition of poly-ubiquitinated proteins (Seibenhener et al., 2004). We noticed that small aggregates formed by the truncated ZZUBA or UBA proteins co-localized with the aggregates formed by recombinant ubiquitin linked to YFP (Ub-VV-YFP). Such co-localization was not observed for truncated Joka2 lacking UBA but possessing PB1 domain. Our data proved that co-localization of Joka2 with ubiquitin linked to YFP is dependent upon the presence of C-terminal UBA domains but not the N-terminal PB1 domain. Therefore, we conclude that at least one of UBA domains of Joka2 (presumably UBA2) is necessary of binding of polyubiquitin aggregates.

**Figure 9:**
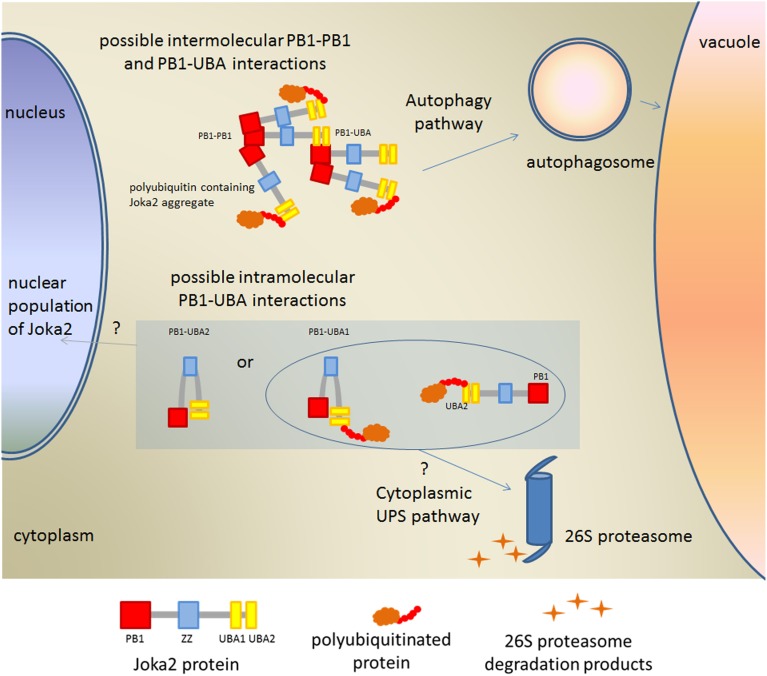
**Model explaining possible PB1-UBA interactions detected in this work**. Involvement of Joka2 in targeting of the ubiquitinated proteins into the cytoplasmic or nuclear Ubiquitin-Proteasomal System (UPS) is only hypothetical.

We have demonstrated that the specific protein-protein interaction between PB1 and at least one of UBA domain of Joka2 is possible. Such interaction was only hypothesized for other selective autophagy cargo receptors due to the high similarity in domain architecture between, for example, p62 and Dsk2 or Rad23 (Su and Lau, 2009). Our data indicated that interaction between PB1 and at least one of UBA domains takes place *in vivo* and that it is much weaker than PB1-PB1 interaction. Interestingly, such PB1-UBA interaction was only observed in aggregates despite the fact that both truncated proteins were spread in the whole cytoplasm. Also for PB1-PB1 interaction the fluorescent signal was observed mostly in aggregates. This conclusion is supported by the observation that detection of the PB1-PB1 interaction in the cytoplasmic non-aggregated fractions of PB1 proteins was possible only in one combination of the vectors. Therefore, we conclude that PB1-PB1 and PB1-UBA interactions take place mainly in aggregates. Aggregates formation is a consequence of self oligomerization of Joka2 and both types of interactions are necessary for multimerization of Joka2 in poly-ubiquitin-containing aggregates (Figure 9).

Interestingly, since the aggregates of p62 have been reported to contain proteasomal components (Seibenhener et al., 2004), it is worth to speculate that Joka2 may interact with proteasomal subunits. Such interaction was previously determined for p62 (Babu et al., 2005; Geetha et al., 2008). The structural similarity of the PB1 domain from Joka2 to the PB1 domain from p62 as well as to the UbL domain, let us hypothesize that Joka2 upon binding of poly-ubiquitinated substrates via one of its C-terminally located UBA domains could bring them directly to proteasome by the presumed direct contact of N-terminal PB1 domain with proteasomal subunits. Thus, it is tempting to speculate that Joka2, similarly to p62 (Seibenhener et al., 2004; Babu et al., 2005; Geetha et al., 2008), could be involved in shuttling of substrates for degradation between UPS and autophagy machinery (Figure 9).

### Conflict of interest statement

The authors declare that the research was conducted in the absence of any commercial or financial relationships that could be construed as a potential conflict of interest.

## Supplementary material

Figure S1 **Graphical illustration of Joka2 and its truncated forms used in this study**. The proteins and domains are drawn to scale. See text for details and domains description.

Figure S2 **Cycling of Joka2-YFP and PB1-YFP between cytoplasm and nucleus is inhibited by LMB treatment**.

Figure S3 **Subcellular localization analysis of fluorescent signal in cells of transgenic tobacco line AB5 expressing free GFP protein treated (+LMB) and not treated (−LMB) with the inhibitor of nuclear export**. No change in fluorescent protein localization could be noticed regardless from LMB treatment. Arrows indicate nuclei.

Figure S4 **Schematic illustration of binary vectors used for BiFC assay**.

Figure S5 **The typical examples of negative controls for BiFC assay**.
