# Supplementary material for: Significant role of PB1 and UBA domains in multimerization of Joka2, a selective autophagy cargo receptor from tobacco
Source: Front Plant Sci. 2014 Jan 31;5:13. doi: 10.3389/fpls.2014.00013 (PMC3907767; doi:10.3389/fpls.2014.00013)

SUPPLEMENTARY MATERIAL

Significant role of PB1 and UBA domains in multimerization of Joka2, a selective autophagy cargo receptor from tobacco

Katarzyna Zientara-Rytter, Agnieszka Sirko*Institute of Biochemistry and Biophysics, Polish Academy of Sciences, ul. Pawinskiego 5a 02-106 Warsaw, Poland

Correspondence

Prof. Agnieszka SirkoInstitute of Biochemistry and BiophysicsPolish Academy of Sciencesul. Pawinskiego 5A02-106 WarsawPolandasirko@ibb.waw.pl

Supplementary Figure 1.

Graphical illustration of Joka2 and its truncated forms used in this study. The proteins and domains are drawn to scale. See text for details and domains description.

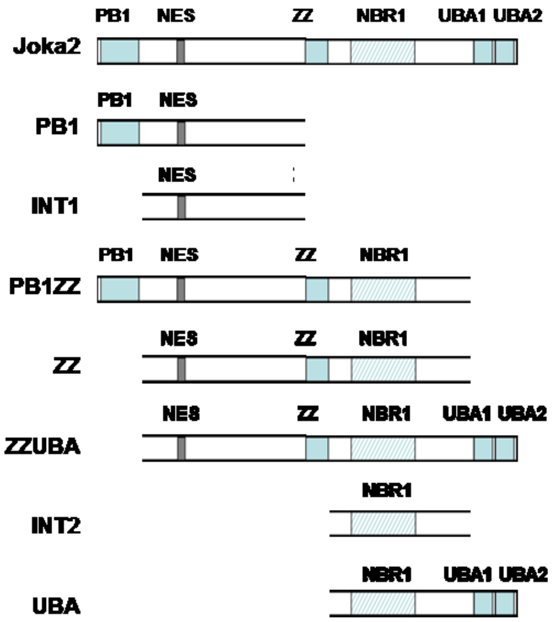

Supplementary Figure 2.

Cycling of Joka2-YFP and PB1-YFP between cytoplasm and nucleus is inhibited by LMB treatment.

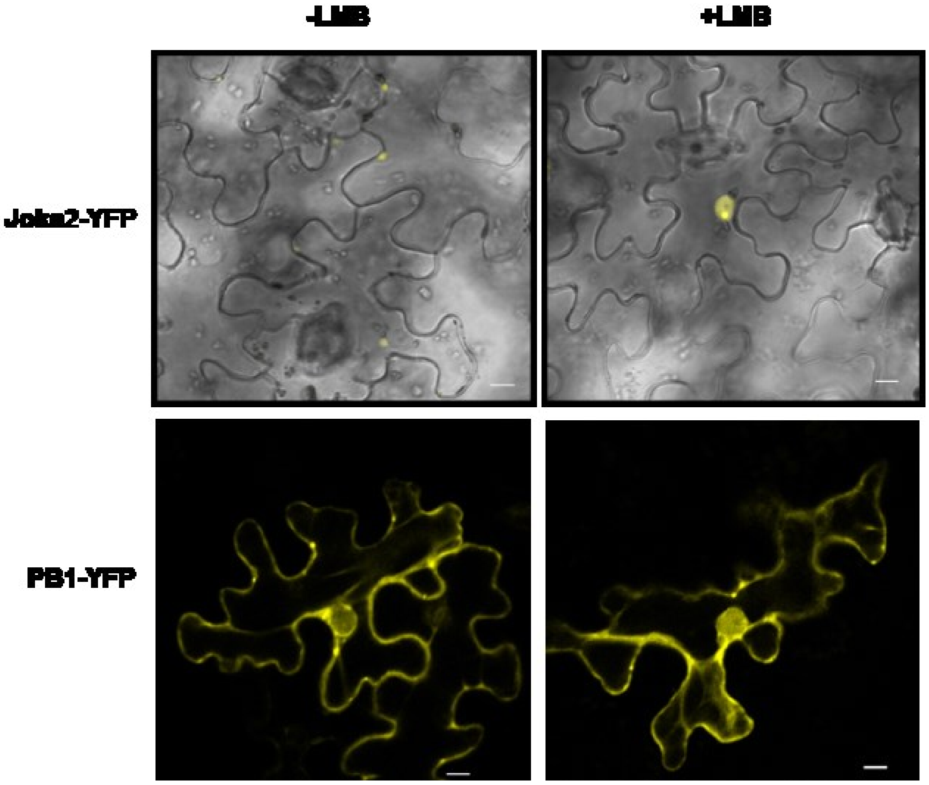

Supplementary Figure 3.

Subcellular localization analysis of fluorescent signal in cells of transgenic tobacco line AB5 expressing free GFP protein treated (+LMB) and not treated (-LMB) with the inhibitor of nuclear export. No change in fluorescent protein localization could be noticed regardless from LMB treatment. Arrows indicate nuclei.

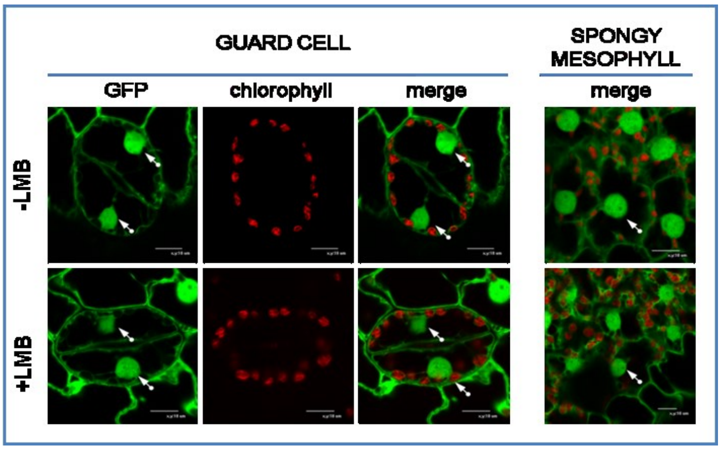

Supplementary Figure 4.

Schematic illustration of binary vectors used for BiFC assay.

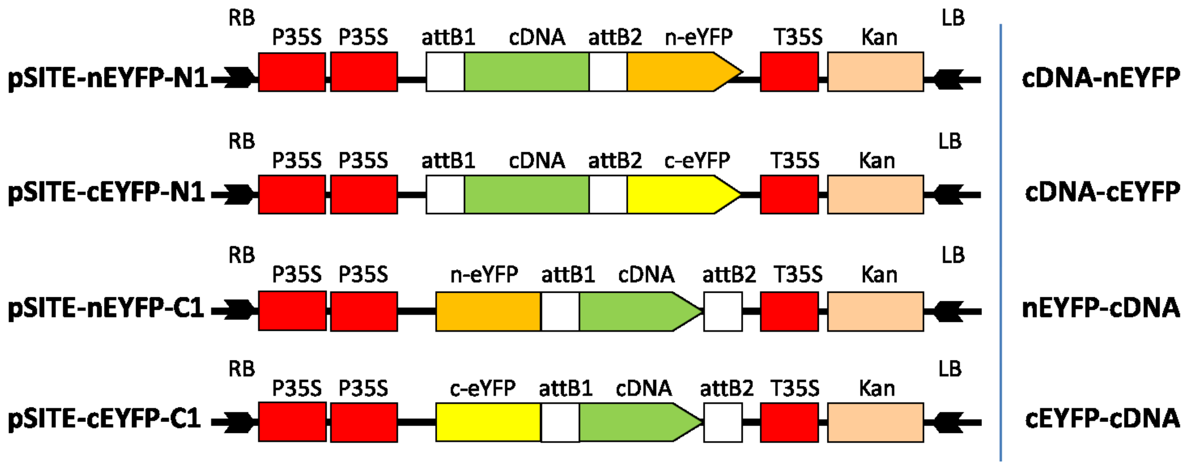

Supplementary Figure 5.

The typical examples of negative controls for BiFC assay.

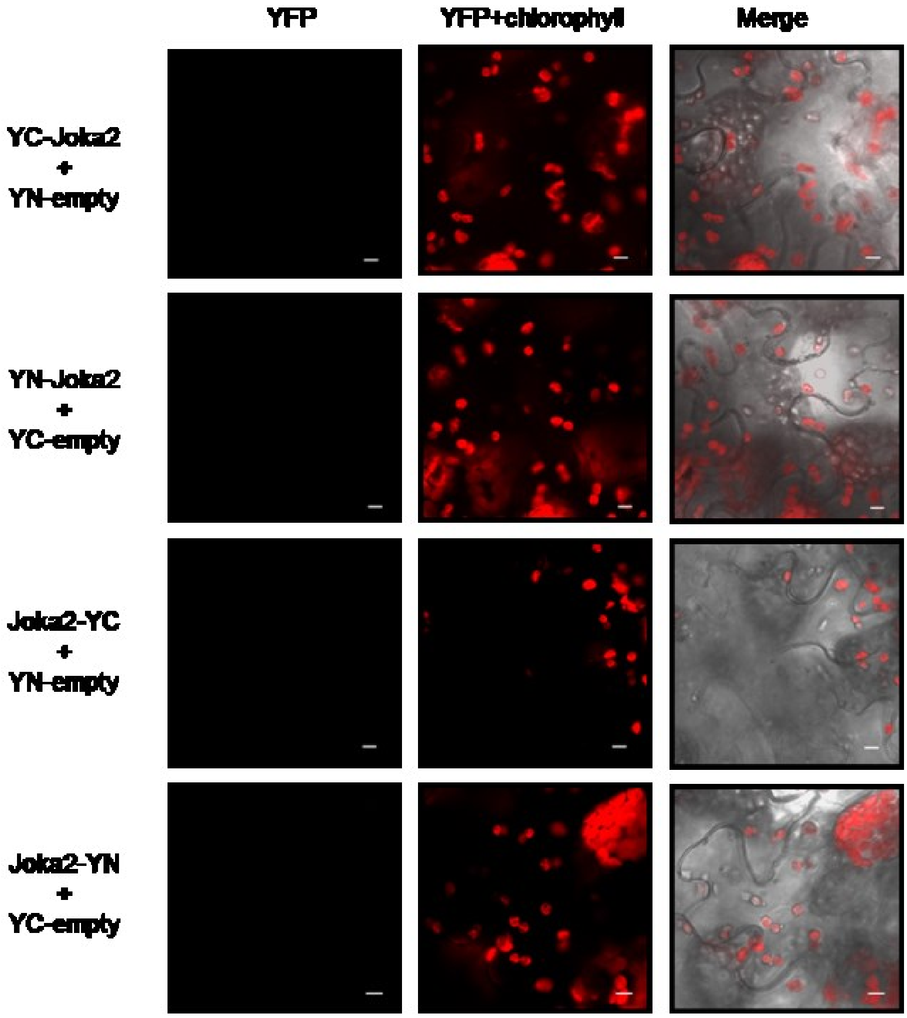

Supplement: Figure S1 — Graphical illustration of Joka2 and its truncated forms used in this study. The proteins and domains are drawn to scale. See text for details and domains description. [file Presentation1.PDF]
